# Comparative analysis of the *Streptococcus pneumoniae* competence development *in vitro* versus *in vivo* during pneumonia-derived sepsis

**DOI:** 10.3389/fmicb.2025.1540511

**Published:** 2025-01-28

**Authors:** Sook Yin Chong, Shi Qian Lew, Tauqeer Alam, Christopher A. Gaulke, Gee W. Lau

**Affiliations:** Department of Pathobiology, University of Illinois at Urbana-Champaign, Urbana, IL, United States

**Keywords:** *Streptococcus pneumoniae*, competence development, pneumonia-derived sepsis, *in vitro* versus *in vivo* gene expression, breach of alveolar-capillary barrier

## Abstract

**Introduction:**

The *Streptococcus pneumoniae* (pneumococcus) competence regulon is well-known for regulating genetic transformation but is also important for virulence. Some pneumococcal strains can enter a transient competent state for genetic transformation in an optimized competence-inducing medium when the threshold level of the peptide pheromone competence stimulating peptide is attained; upregulating the expression of three distinct phases of “early”, “late” and “delayed” competence genes. Recently, we discovered that pneumococcus can naturally enter a prolonged competent state during acute pneumonia in mice. However, mechanisms driving competence development during host infection are rarely examined, and a direct comparison between *in vitro* and *in vivo* competence induction has not been performed.

**Methods:**

We conducted a comparative gene expression analysis of pneumococcal competence development *in vitro* versus *in vivo* during pneumonia-derived sepsis in mice. We examined existing RNA-Seq data and performed validation using RNA obtained from an independent replicate experiment.

**Results and discussion:**

Our analysis revealed both similarities and differences in the expression of “early”, “late”, and “delayed” competence between *in vitro* versus during pneumonia-derived sepsis. Our results may reveal new aspects of pneumococcal competence biology.

## Introduction

*Streptococcus pneumoniae* (pneumococcus) is an important human pathogen that causes community-acquired pneumonia, pneumonia-derived sepsis (pneumonic sepsis) and associated multi-organ dysfunction, otitis media, and meningitis among young children and the elderly (Olson and Davis, 2020; Gadsby and Musher, 2022; Feldman and Anderson, 2016). Pneumococcal infections are linked to high rates of morbidity and mortality. The pneumococcal capsule is a major virulence factor accounting for more than 100 serotype diversity (Zafar et al., 2017; Paton and Trappetti, 2019; Geno et al., 2015). Vaccination remains the best practice to prevent pneumococcal diseases. However, full coverage of ≥100 serotypes with currently licensed 23-valent capsular polysaccharide vaccine (Pneumovax 23) and conjugated vaccines (PCV7, 10, 15, 20), is not feasible (Feldman and Anderson, 2014; Jaiswal et al., 2014; Mehr and Wood, 2012). The highly recombinogenic nature of pneumococcus has led to the emergence of vaccine escape strains through “capsule switching” to non-vaccine serotypes (Mehr and Wood, 2012; Manna et al., 2022; Feikin et al., 2013). Additionally, the increasing prevalence of antibiotic-resistant pneumococcal strains has impeded the momentum for eradicating pneumococcal diseases (Nikolaou et al., 2020; Li et al., 2023; Johnson et al., 2024), leading to increased healthcare costs.

Another major challenge in combatting pneumococcal diseases is its ability to induce natural competence. Pneumococcus develops a competent state by which it actively takes up exogenous DNA into its cells for genetic transformation (Griffith, 1928; Salvadori et al., 2019; Claverys et al., 2009), as well as regulation of virulence processes (Lin et al., 2016; Zhu et al., 2015; Lau et al., 2001). For decades, the molecular mechanisms of pneumococcal competence development have been intensively investigated using the capsule-deficient strains derived from the serotype 2 strain D39 (Griffith, 1928), including CP1200, CP1250, and R6, as well as its hypercompetent lineages R800 and Rx, under specific *in vitro* culture conditions (e.g., using the competence-optimized C + Y medium) (Stevens et al., 2011; Gagne et al., 2013; Mirouze et al., 2013; Guiral et al., 2006; Bergé et al., 2017). In contrast, D39 could only enter the competent state in the Todd Hewitt supplemented with yeast extract (THY) broth when exogenously provided with the quorum sensing peptide pheromone called competence stimulating peptide (CSP) (Lin et al., 2020). The competent state develops naturally in response to the threshold accumulation of CSP encoded by the *comC* gene (Havarstein et al., 1995; Pestova et al., 1996; Håvarstein et al., 1996). The pre-CSP is processed into a mature peptide and secreted through the ComAB transporters. When extracellular CSP reaches a critical concentration, it binds to ComD and activate the two-component histidine kinase-response regulator signal transduction system (TCSTS) ComDE (Martin et al., 2013). The binding of CSP to ComD results in the phosphorylation of ComE. Phosphorylated ComE acts as a transcription factor and binds to *com*-boxes on the promoter of some *com* genes to activate their transcription, including *comAB*, *comCDE*, and *comX1comX2* operons, creating a positive feedback loop which amplifies the competence signal.

ComDE triggers a short transient burst of the competent state that augments the expression of three distinct but temporally overlapping phases of “early,” “late,” and “delayed” competence (*com*) genes during competence development (Rimini et al., 2000; Peterson et al., 2004). The expression of early *com* genes *comABCDE* is pivotal in initiating the transition of pneumococcal cells into the competent state and results in the expression of the alternative σ factor ComX, encoded by the two identical copies of the early *com* genes *comX1* and *comX2* (Luo et al., 2003; Luo and Morrison, 2003; Piotrowski et al., 2009; Lee and Morrison, 1999). ComX activates the transcription of late *com* genes, facilitating processes such as DNA uptake and recombination (Peterson et al., 2004; Luo and Morrison, 2003; Weyder et al., 2018), and enhances virulence mechanisms, partially via fratricide (Havarstein et al., 2006; Steinmoen et al., 2002; Eldholm et al., 2010) mediated release of pneumolysin (Zhu et al., 2015; Brown et al., 2014; Alhamdi et al., 2015; Price and Camilli, 2009; Zafar et al., 2017). These include genes encoding components of the DNA binding type IV pilus (*comG* operon), the DNA receptor (*comEA*), the DNA translocase (*comFA*) (Laurenceau et al., 2015), and DNA processing and recombination proteins (*ssbB*, *dprA*, and *recA*) (Mortier-Barrière et al., 2007; Attaiech et al., 2011), which collectively facilitate DNA transfer across the cellular membrane and its integration into the genome. Although the regulation of delayed *com* genes is less well understood, they are thought to play a significant role in the fitness of *S. pneumoniae* during infection and associated with stress response mechanisms (Rimini et al., 2000; Peterson et al., 2004; Dagkessamanskaia et al., 2004).

The spontaneously developed transient competent state observed *in vitro* for *S. pneumoniae* lasts for 30–40 min, after which it is rapidly shut off (Rimini et al., 2000; Peterson et al., 2004; Peterson et al., 2000). In a previously published DNA microarray study using *S. pneumoniae* TIGR4 strain, Peterson et al. (2004) reported that the provision of CSP induces the expression of early *com* genes rapidly, reaching peak expression between 7.5 and 10 min. A delay of approximately 5 min was observed for the late *com* genes to reach their maximum expression. Additionally, the mRNA levels for delayed *com* genes exhibited a continual increase and reached their peak by the first 15–17 min post-CSP treatment. However, in our recent *in vivo* imaging system (IVIS)-based live imaging study during pneumonic sepsis in CD-1 mice using a D39 reporter strain harboring a firefly luciferase transcriptionally fused to the late competence *ssbB* gene (D39-*ssbB*-*luc*) (Lin and Lau, 2019), D39 was able to enter the competent state naturally without the exogenously provided CSP1 (Lin et al., 2020). The competent state during the acute lung infection was prolonged and persistent and occurred only after 20–24 h post-infection (hpi). This was rapidly followed by the breach of the alveolar-capillary barrier, systemic bacteremia and sepsis. The naturally developed competent state was maintained throughout the septic stage until moribund mice were euthanized (Lin et al., 2020). In our most recent study using a more sensitive RNA-Seq approach during pneumonic sepsis, we found that the expression of the early and late *com* genes was elevated as early as 12-hpi, and revealed a temporal period of adaptation to the host lung environment due to metal scarcity before the development of natural competence (Oh et al., 2024).

Therefore, in this study, we aim to unravel the differences in competence-responsive genes *in vitro* versus during pneumonia-derived sepsis. We comprehensively analyzed the existing pneumonia-derived sepsis RNA-Seq data (Oh et al., 2024) along with *in vivo* gene expression data from a repeated mouse pneumonic sepsis experiment to compare the expression of selective “early,” “late,” and “delayed” *com* genes against the expression of these genes *in vitro* during CSP induced competence development. Our analysis clearly illustrates the similarities and differences between the expression of *com* genes *in vitro* versus *in vivo* during pneumonia-derived sepsis.

## Materials and methods

### Bacterial cultures

The serotype 2 parental wild-type *S. pneumoniae* strain D39 and the firefly luciferase reporter derivative D39-*ssbB*-*luc* were previously described (Lin et al., 2020; Lin and Lau, 2019; Oh et al., 2024). For the *in vitro* study, the frozen stock of D39 was plated on Todd-Hewitt Yeast (THY) agar (#249240 Becton Dickinson) and incubated overnight at 37°C with 5% CO_2_. After overnight growth, a fresh D39 single colony was picked and cultured in the THY broth to mid-log phase of OD_600_ of 0.2. For competence development, exogenous CSP1 (100 ng mL^−1^) was added, and samples were collected at every 10 min interval for 40 min, followed by RNA isolation.

For *in vivo* studies, the D39 *ssbB*-*luc* strain was plated on a THY agar plate supplemented with 5 μg mL^−1^ chloramphenicol (#C0378, Sigma, St. Louis, MO) and incubated overnight at 37°C with 5% CO_2_. To mitigate inaccuracies in dosing caused by the tendency of pneumococci to form long chains during culture in THY broth (Lin et al., 2020), we employed a modified preparation method. A single colony was selected and grown in THY broth until it reached an OD_600_ of 0.2. The culture was then spread on Columbia agar supplemented with 5% sheep blood (#R01217, Thermo Scientific, Waltham, MA), which promotes diplococci formation, similar to the cellular morphology observed in clinical specimens. This approach ensured more precise bacterial enumeration for subsequent mouse inoculation. After 5 h, the bacteria cells were scrapped off from the blood agar surface and collected. The harvested cells were then washed thrice with 0.9% saline and diluted to the desired concentration for mouse infection.

### Ethics statement

This study was conducted in strict accordance with the recommendations in the Guide for the Care and Use of Laboratory Animals by the National Institutes of Health (NIH). The protocols (#21086 and 24078) were approved by the Institutional Animal Care and Use Committee at the University of Illinois at Urbana-Champaign.

### Mouse acute pneumonia infection and IVIS live imaging

CD-1 mice (7 weeks old, males and females, groups of 5) were purchased from Charles River Laboratories (Boston, MA). The mice were anesthetized with isoflurane and intranasally administered with 50 μL of 10^7^ colony-forming units (CFU) of D39-*ssbB*-*luc*. The inoculum dose was validated by serial dilution plating on THY agar supplemented with 5 μg mL^−1^ chloramphenicol. Mouse live imaging was carried out by using an IVIS SpectrumCT imaging system (Perkin-Elmer, Waltham, MA). Briefly, mice were anesthetized with 3% isoflurane in an induction chamber followed by intraperitoneal injection of D-luciferin potassium 100 mg kg^−1^ (LUCK; GoldBio, St. Louis, MO) dissolved in DPBS for 10 min to allow systemic spread of the substrate before imaging. Luminescence images were captured with the following settings: binning factor = 8, *f*-number = 1, field of view = 25.4 cm, luminescent exposure time = 60s. The acquired images were analyzed by Living Image Software (Perkin-Elmer). Mice were euthanized at four different time points (T1 = 0-hpi, T2 = 12-hpi, T3 = 24-hpi, and T4 = >40-hpi), and lungs were harvested and processed for total RNA extraction.

### RNA isolation

For *in vitro* studies, pneumococcal bacteria were harvested mid-log phase at the OD_600_ of 0.2, followed by the addition of the RNAprotect Bacteria Reagent (#76506 from Qiagen, Hilden, Germany) according to manufacturer’s instructions and stored at −80°C until use. For subsequent RNA isolation, the bacterial pellets were thawed to room temperature, resuspended in fresh TRIzol reagent (#15596026 from Invitrogen, Carlsbad, CA) and incubated at room temperature for 5 min, and subjected to mechanical disruption with 0.15 mm Zirconium Beads (#ZrOB051, Next Advance, Raymertown, NY) for 10 min using the Bullet Blender Homogenizer (Next Advance, Raymertown, NY). Next, chloroform was added to the samples and centrifuged at 12,000 × *g* for 15 min at 4°C and the aqueous phase was collected for subsequent RNA purification using the Direct-zol RNA Miniprep Plus kit (#R2070, Zymo Research, Irvine, CA) according to manufacturer’s instructions. For *in vivo* studies, the harvested lungs were chopped into smaller pieces and stored at −80°C until further use. Frozen lung sample was carefully homogenized with −80°C pre-chilled pestle and mortar until fine powder is obtained. Next, the sample was further homogenized with a total of 4 mL of TRIzol reagent and 1 g of aluminium oxide powder (#265497, Sigma-Aldrich, St. Louis, MO) for 10 min. Liquid nitrogen was added periodically to prevent RNA from degradation and the samples were transferred to microcentrifuge tubes and centrifuged at 7,600 × *g* for 5 min to remove the aluminium oxide. The supernatant was collected and subjected to additional mechanical disruption with 0.15 mm Zirconium Beads using Bullet Blender Homogenizer for 10 min to further lyse any residual bacteria. Next, the homogenized sample was centrifuged at 12,000 × *g* for 15 min after the addition of chloroform for phase separation. An adequate amount of TRIzol reagent can be added if phase inversion was observed followed by re-centrifugation. The colourless upper aqueous phase was collected for subsequent RNA isolation according to manufacturer’s instructions.

### cDNA preparation and qPCR

For cDNA preparation, 1 μg of total pneumococcal RNA was reversely transcribed using the SuperScript IV First-Strand Synthesis Kit (#1809150 from Invitrogen, Carlsbad, CA) with random primers according to the manufacturer’s instructions. The qPCR reaction was performed using 10 μL of PowerUp SYBR Green Master Mix (#A25742 from Applied Biosystems, Waltham, MA), 0.4 μL (10 μM) of forward and reverse primers, 6 μL of cDNA (diluted 1:4 for *in vivo* samples and 1:9 for *in vitro* samples), and water added to a total volume of 20 μL. qPCR reactions were performed using a QuantStudio 3 Real-Time PCR System (Applied Biosystems, Waltham, MA) with the following cycling conditions: 95°C for 10 min, 40 cycles of 95°C for 15 s and 60°C for 1 min. The sequences of various primers used for all *com* genes are listed in Supplementary Table S2. The *gyrA* was used for internal control as previously published by others (Pettigrew et al., 2014; Manna et al., 2018; Minhas et al., 2020).

### RNA-Seq analysis

**Figure 1:**
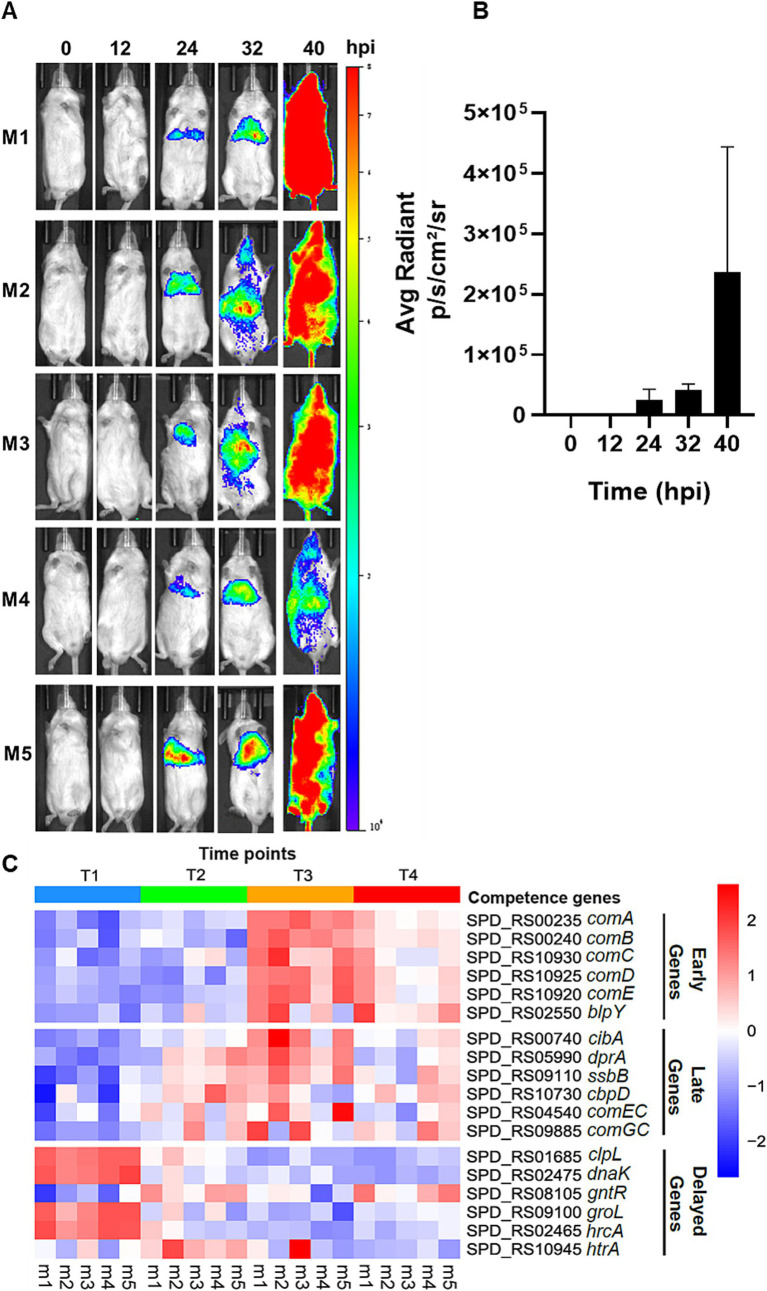
Live imaging of pneumonic sepsis mice and heatmap of gene expression by RNA-Seq. **(A)** Representative images of mice with naturally developed competent state during pneumonia-derived sepsis. CD-1 mice (*n* = 5) were intranasally inoculated with 10^7^ CFU of D39 *ssbB*-*luc* and imaged using an IVIS SpectrumCT imaging system by detecting the bioluminescent signals after subcutaneous injection with 50 μL of 20 mg mL^−1^ D-luciferin potassium at T1 = 0-hpi, T2 = 12-hpi, T3 = 24-hpi, and T4 = >40-hpi. **(B)** Quantification of bioluminescent signals in **(A)** by the Living Image Software (Perkin-Elmer). **(C)** The heatmap represents the expression profile of competence-responsive genes in all five mice at T1, T2, T3, and T4. The squares are colored based on *z-*score, with red color representing upregulation in gene expression and blue color representing downregulation in gene expression.

**Figure 2:**
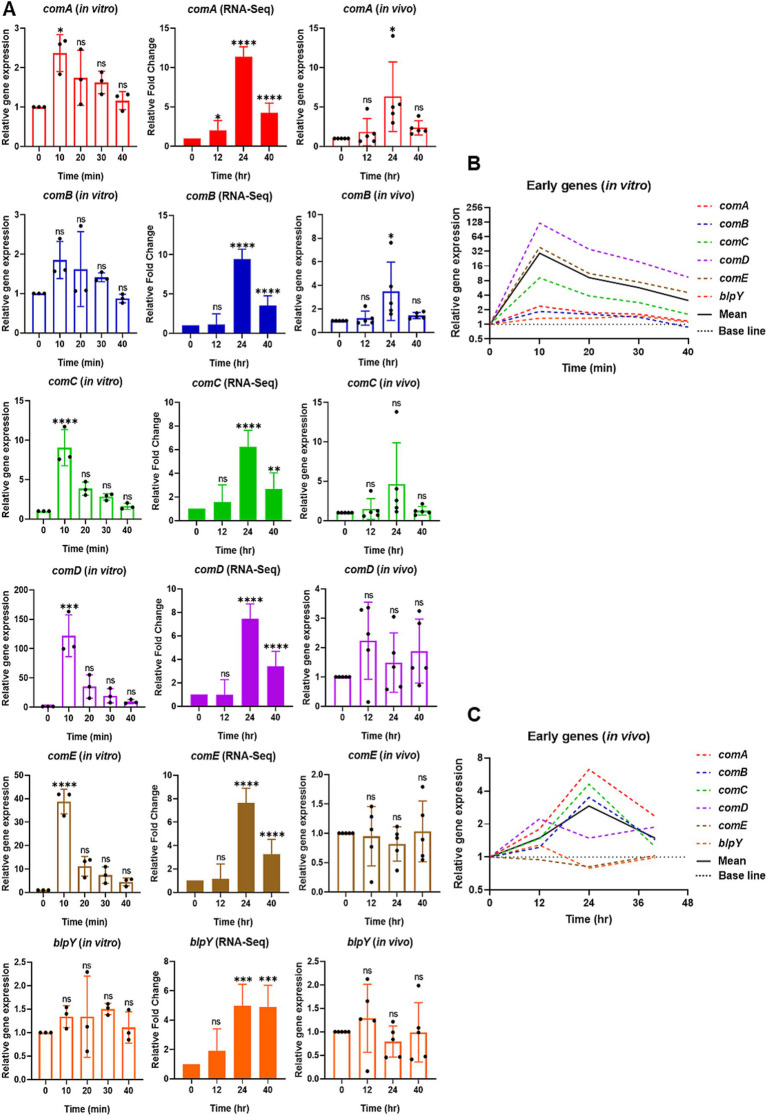
Relative expression of selective early competence (*com*) responsive genes *in vitro* by qPCR (left), RNA-Seq (middle), and reproducibility validation of *in vivo* RNA-Seq results by qPCR (right). **(A)** For *in vitro*, the bar graphs show the relative gene expression of early *com* genes: *comA*, *comB*, *comC*, *comD*, *comE*, and *blpY*. The bacterial cells were collected at 0 min at the mid-log phase of OD_600_ of 0.2. After CSP1 (100 ng/mL) addition, bacterial samples were collected at 10-min intervals for 40 min followed by RNA extraction. The relative expression of each gene at Time 0 min (baseline control without addition of CSP1) was set to one. Bars represent the mean of three biological replicates from three independent experiments with standard deviation. For RNA-Seq and reproducibility validation of *in vivo* RNA-Seq using qPCR, the bar graphs show the relative fold change of early *com* genes at T1 = 0-hpi, T2 = 12-hpi, T3 = 24-hpi, and T4 = >40-hpi. Bars represent the mean of five biological replicates (*n* = 5 infected CD-1 mouse lungs) with their corresponding standard error. The relative expression of each gene at Time 0-hpi was set to one and *gyrA* gene was used as the reference gene for data normalization. ^*^p < 0.05, ^**^p < 0.01, ^***^p < 0.001, and ^****^p < 0.0001 when compared to Time 0 min (*in vitro*) and Time 0-hpi (*in vivo*) using the Student’s *t*-test with Holm–Bonferroni correction. The relative expression of all the selective early *com* genes **(B)** *in vitro* and **(C)** validation of *in vivo* RNA-Seq using qPCR was plotted in one graph, with mean expression shown in a solid black line.

**Figure 3:**
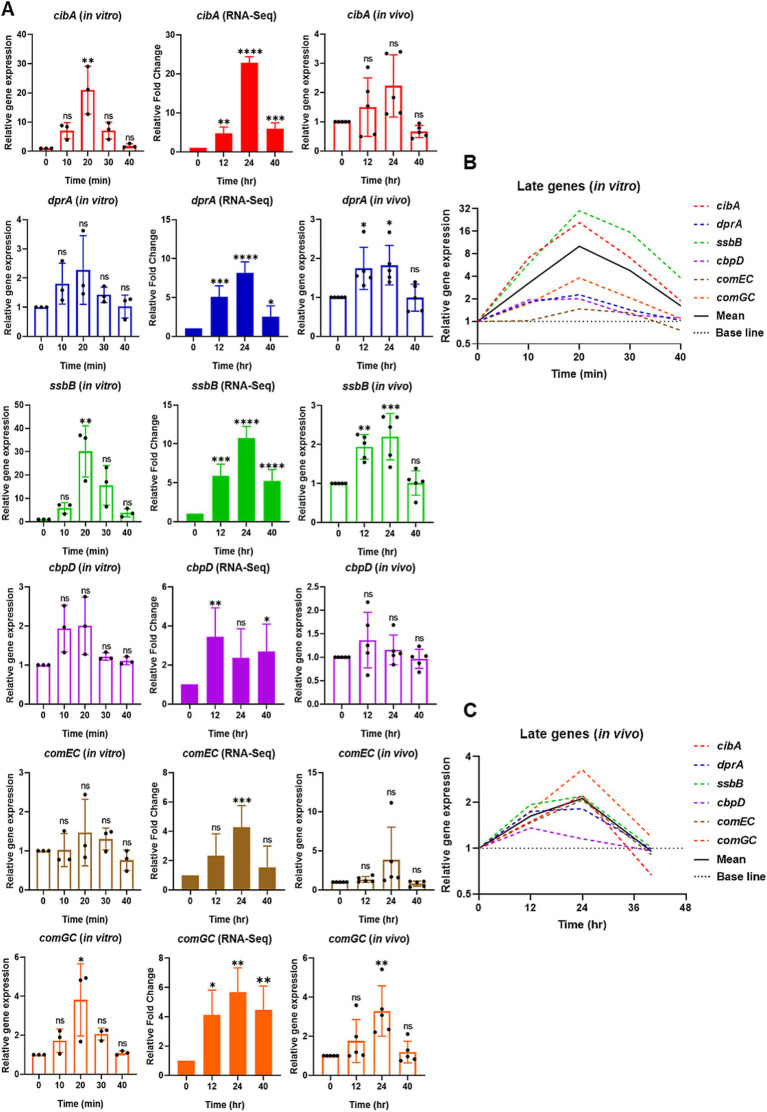
Relative expression of selective late competence (*com*) responsive genes *in vitro* by qPCR (left), RNA-Seq (middle), and reproducibility validation of *in vivo* RNA-Seq results by qPCR (right). **(A)** For *in vitro*, the bar graphs show the relative gene expression of late *com* genes: *cibA*, *dprA*, *ssbB*, *cbpD*, *comEC*, and *comGC*. The bacterial cells were collected at 0 min at the mid-log phase of OD_600_ of 0.2. After CSP1 (100 ng/mL) addition, bacterial samples were collected at 10-min intervals for 40 min followed by RNA extraction. The relative expression of each gene at Time 0 min (baseline control without addition of CSP1) was set to one. Bars represent the mean of three biological replicates from three independent experiments with standard deviation. For RNA-Seq and reproducibility validation of *in vivo* RNA-Seq using qPCR, the bar graphs show the relative fold change of late *com* genes at T1 = 0-hpi, T2 = 12-hpi, T3 = 24-hpi, and T4 = >40-hpi. Bars represent the mean of five biological replicates (*n* = 5 infected CD-1 mouse lungs) with their corresponding standard error. The relative expression of each gene at Time 0-hpi was set to one and *gyrA* gene was used as the reference gene for data normalization. ^*^p < 0.05, ^**^p < 0.01, ^***^p < 0.001, and ^****^p < 0.0001 when compared to Time 0 min (*in vitro*) and Time 0-hpi (*in vivo*) using the Student’s *t*-test with Holm–Bonferroni correction. The relative expression of all the selective late *com* genes **(B)** *in vitro* and **(C)** validation of *in vivo* RNA-Seq using qPCR was plotted in one graph, with mean expression shown in a solid black line.

**Figure 4:**
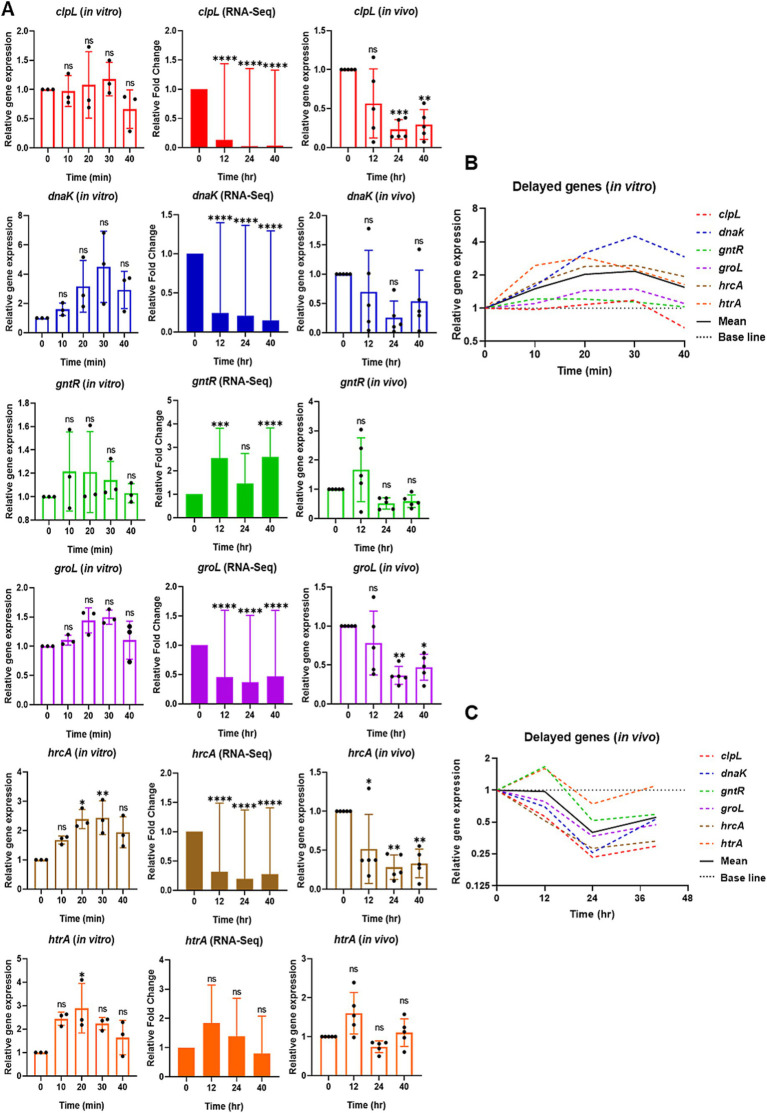
Relative expression of selective delayed competence (*com*) responsive genes *in vitro* by qPCR (left), RNA-Seq (middle), and reproducibility validation of *in vivo* RNA-Seq results by qPCR (right). **(A)** For *in vitro*, the bar graphs show the relative gene expression of delayed *com* genes: *clpL*, *dnaK*, *gntR*, *groL*, *hrcA*, and *htrA*. The bacterial cells were collected at 0 min at the mid-log phase of OD_600_ of 0.2. After CSP1 (100 ng/mL) addition, bacterial samples were collected at 10-min intervals for 40 min followed by RNA extraction. The relative expression of each gene at Time 0 min (baseline control without addition of CSP1) was set to one. Bars represent the mean of three biological replicates from three independent experiments with standard deviation. For RNA-Seq and reproducibility validation of *in vivo* RNA-Seq using qPCR, the bar graphs show the relative fold change of delayed *com* genes at T1 = 0-hpi, T2 = 12-hpi, T3 = 24-hpi, and T4 = > 40-hpi. Bars represent the mean of five biological replicates (*n* = 5 infected CD-1 mouse lungs) with their corresponding standard error. The relative expression of each gene at Time 0-hpi was set to one and *gyrA* gene was used as the reference gene for data normalization. ^*^p < 0.05, ^**^p < 0.01, ^***^p < 0.001, and ^****^p < 0.0001 when compared to Time 0 min (*in vitro*) and Time 0-hpi (*in vivo*) using the Student’s *t*-test with Holm–Bonferroni correction. The relative expression of all the selective delayed *com* genes **(B)** *in vitro* and **(C)** validation of *in vivo* RNA-Seq using qPCR was plotted in one graph, with mean expression shown in a solid black line.

**Figure 5:**
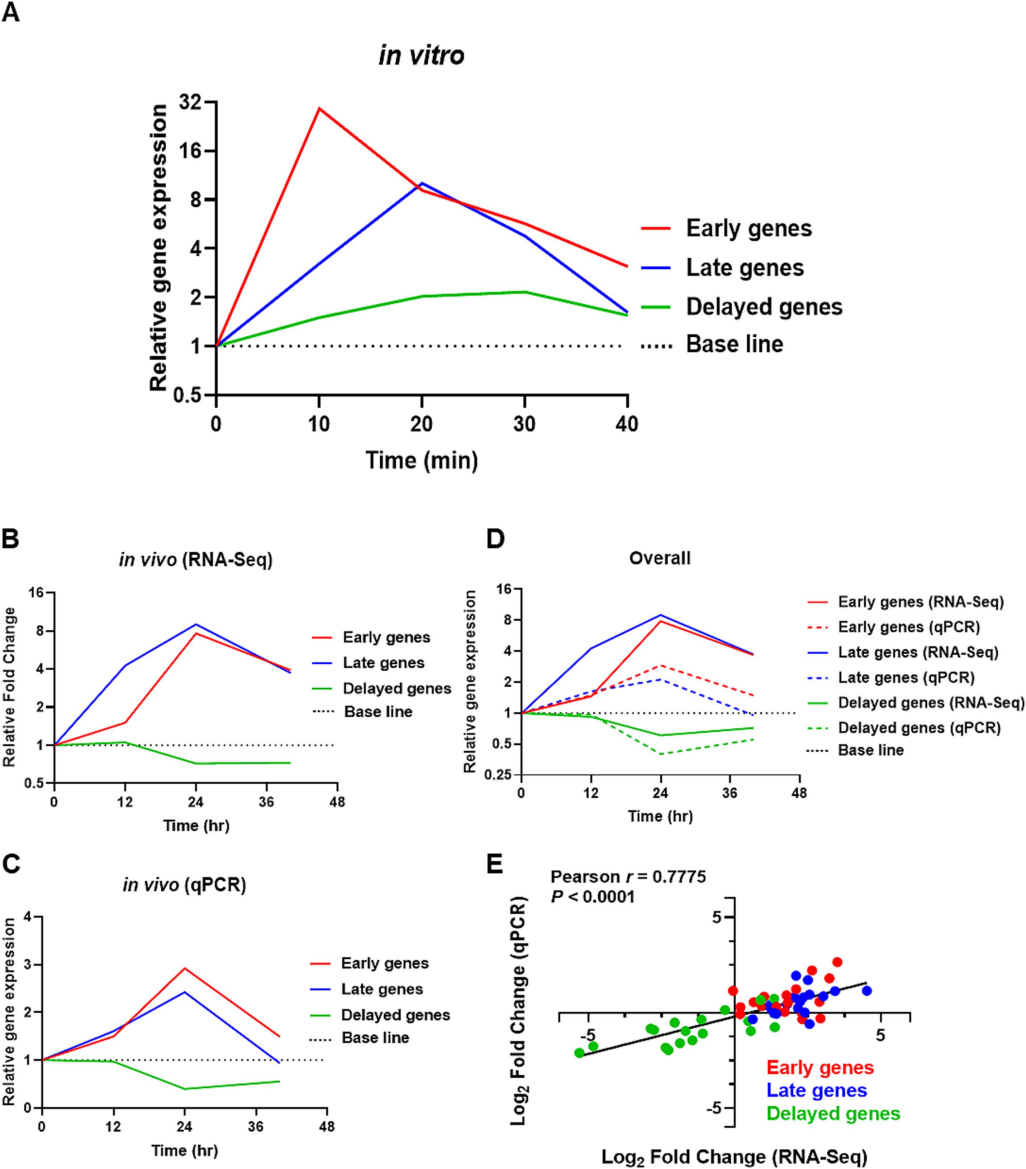
*In vitro* qPCR and reproducibility validation of *in vivo* RNA-Seq transcriptomic data with qPCR. **(A)** Average expression of selective *com* responsive genes *in vitro* by qPCR, with red = early *com* genes, blue = late *com* genes, and green = delayed *com* genes. The base line was set to one. **(B,C)** Shows the average expression levels of competence-responsive genes *in vivo* from RNA-Seq and validation from qPCR, respectively. **(D)** Show the average gene expression values of *com* genes obtained from RNA-Seq (solid line) and qPCR (dotted line). **(E)** Log_2_ fold changes in gene expression from qPCR were plotted against RNA-Seq transcriptomic data for all 18 *com* genes. Each data point within the dataset represents the log_2_ fold change at 12, 24, and 40-hpi relative to 0-hpi, resulting in a total of 54 plotted values. Red dots represent early *com* genes, blue dots represent late *com* genes, and green dots represent delayed *com* genes. A high degree of correlation was observed with Pearson *r* = 0.7775 (*p* < 0.0001). *X*-axis: Log_2_ fold change (RNA-Seq). *Y*-axis: Log_2_ fold change (qPCR).

The *in vivo* RNA-Seq study, including mouse infection, RNA purification, library preparation and sequencing was previously published (Oh et al., 2024). The raw FASTQ reads have been submitted to the NCBI’s Sequence Read Archive (SRA) under the Bioproject ID PRJNA1204359 and BioSample IDs SAMN35715190–SAMN35715209, and were used for analysis in Figures 1C, 2A, 3A, 4A, 5B,D,E.

### Statistical analysis

Statistical analyses were performed using R studio software version 4.3.2 and GraphPad Prism statistical software package version 8.0.2. The statistical differences when comparing between two groups were determined by a paired Student’s *t-*test. Statistical significance was set at ^*^p < 0.05, ^**^p < 0.01, ^***^p < 0.001, and ^****^p < 0.0001, or ns (not significant).

## Results

### *Streptococcus pneumoniae* induces a prolonged and persistent competent state during pneumonic sepsis

Recently, we have demonstrated that pneumococcus serotype 2 strain D39, serotype 3 strain 0100993, and serotype 4 strain TIGR4 were able to enter a prolonged and persistent competent state naturally without the exogenously provided CSP1 during the acute lung infection (Lin et al., 2020). We attempted to authenticate the pneumococcal competence development in CD-1 mice infected with 10^7^ CFU of D39-*ssbB*-*luc* strain by using an *in vivo* SpectrumCT imaging system (IVIS) based live imaging, as previously described (Lin et al., 2020; Oh et al., 2024). At 0 and 12-hpi, no competence signal was observed in any of the mice, and the competence signal was visible at approximately 24-hpi in the infected lungs (Figure 1A). The competent state intensified significantly during the systemic infection after the breach of the alveolar-capillary barrier, at approximately 32-hpi (e.g., Figure 1A, mice M2 and M3). The competence signal in each mouse persisted beyond 40-hpi and achieved the highest intensity at the moribund stage. Quantification of bioluminescence signals indicates the average radiant intensity at 24-hpi was 2.51 × 10^4^, which increased to 4.18 × 10^4^ at 32-hpi and peaked at >40-hpi with a value of 2.37 × 10^5^ (Figure 1B). Recently, we subjected mouse lungs infected by D39-*ssbB-luc* to RNA-Seq guided by IVIS-based monitoring of the competence development at 0, 12, 24, and at the moribund state >40-hpi. There was significant upregulation of early, late, and some delayed phase competence-specific genes at 12, 24 and 40-hpi, revealing that maintenance of the competence state is important for initial adaptation to the lung environment and the pathogenesis during pneumococcal pneumonic sepsis (Oh et al., 2024). To gain a deeper understanding of the disparities in the competence development between *in vitro* versus during acute pneumonic sepsis, the expression of selective early, late, and delayed *com* genes (Supplementary Table S1) was analyzed by quantitative real-time PCR (qPCR) in the wild-type strain D39 after the induction by CSP1 *in vitro*. The temporal expression profiles of the selective *com* gene from the RNA-Seq is re-presented in the heatmap in Figure 1C.

### Comparative analysis of the pneumococcal early *com* gene expression *in vitro* versus during pneumonic sepsis

First, we focused on selective early *com* genes, including *comA*, *comB*, *comC*, *comD*, *comE*, and *blpY*. The results showed that most of the early genes were rapidly induced to initiate the competent state, reaching their highest expression 10 min after exposure to CSP1. In particular, the expression of *comA*, *comC*, *comD*, and *comE* are significantly upregulated at 10 min post-CSP1 treatment. Although the expression levels of *comB* and *blpY* peaked at 10 min post-induction by CSP1, they were not statistically different from Time 0 min (basal expression) due to large error bars (Figure 2A). However, the average expression of *comB* and *blpY* at 10 min is at least 1.85-fold higher and 1.34-fold higher, respectively, than at Time 0 min. As reported in Kjos et al. (2016) although the expression of *blp* locus-encoded bacteriocins is co-regulated by the competence system and the BlpHR TCSTS (Throup et al., 2000), *blp* genes are expressed at low levels during pneumococcal competence *in vitro*. Overall, the average gene expression of the selective early *com* genes is at their highest expression at 10 min post-induction by CSP1, as shown in the solid black line in Figure 2B.

In our initial effort to understand the dissimilarities in pneumococcal competence development between *in vitro* condition and during pneumonic sepsis, we conducted a comprehensive re-analysis of our previously published RNA-Seq data derived from studies on a mouse model of acute pneumonia-derived sepsis infection by the pneumococcus strain D39 (Oh et al., 2024). While the *in vivo* competence induction has been studied (Oh et al., 2024), a direct comparison to *in vitro* competence induction has not been performed. Therefore, a re-examination of our existing RNA-Seq data set was necessary and specifically, aimed at facilitating an effective comparison between the *in vitro* and *in vivo* scenarios, providing new insights into the competence development process under different environmental conditions. The expression of most of the early *com* genes began to increase at 12-hpi (T2), peaking at 24-hpi (T3), which coincided with the time point when all the infected mice had transitioned into the competent state during acute lung infection. The expression of early *com* genes decreased slightly post 24-hpi, but was maintained well above the baseline levels at 0-hpi (T1; Figure 2A). While *S. pneumoniae* transcribes transient competence-responsive genes *in vitro*, the *in vivo* expression pattern contrasts sharply with a prolonged and persistent state during *in vivo* infection. This extended duration of competence gene expression in the infected lungs indicates that *S. pneumoniae* differentially responds to distinct environmental cues during infection compared to *in vitro* growth.

To validate the reproducibility of the RNA-Seq transcriptomic data, RNA samples from our new pneumonic sepsis experiment were analyzed by qPCR. The qPCR findings revealed that early *com* genes such as *comA*, *comB*, and *comC* exhibited a significant increase in expression levels at 24-hpi compared to levels at 0-hpi. However, due to an extreme outlier at 24-hpi for *comC*, the statistical difference was not significant. The average expression of *comC* at 12-hpi and 24-hpi is at least 1.47-fold and 4.63-fold higher than at 0-hpi, respectively, indicating an increase in gene expression and reaching highest at 24-hpi. Surprisingly, the qPCR results for *comD*, *comE*, and *blpY* did not align with the RNA-Seq data. The RNA-Seq revealed that the expression of *comD*, *comE*, and *blpY* increased by 5-7-fold at 24-hpi (Figure 2A). However, partially due to large error bars, the qPCR results showed that the expression of *comD* only increased by 2.23-fold at 12-hpi and maintained at levels higher than baseline at 24 and 40-hpi, while the expression of *comE* and *blpY* remained near the baseline levels at 12, 24, and >40-hpi. Overall, an average gene expression of the selective early *com* genes displayed their highest expression at 24-hpi, as shown in the solid black line in Figure 2C.

### Comparative analysis of the pneumococcal late *com* gene expression *in vitro* versus during pneumonic sepsis

The initial expression of the early *com* genes is followed by the expression of late *com* genes known to encode functions necessary for DNA uptake and recombination (Rimini et al., 2000; Peterson et al., 2004; Peterson et al., 2000; Johnsborg and Havarstein, 2009). To further elucidate the differences in competence induction between *in vitro* conditions and during pneumonia-derived sepsis, we conducted a comparative analysis of selective late *com* genes, including *cibA*, *dprA*, *ssbB*, *cbpD*, *comEC*, and *comGC* using qPCR. The expression of *cibA*, *ssbB*, and *comGC* genes exhibited markedly higher expression levels at 20 min after CSP1 treatment *in vitro* (Figure 3A). Although no statistically significant differences were observed in the expression levels of *dprA*, *cbpD*, and *comEC* compared to baseline (Time 0 min), a notable peak in expression was detected at 20 min post-CSP1 exposure. The expression of *dprA* increased 1.80-fold and 2.28-fold at 10 and 20 min, respectively, relative to baseline. Similarly, *cbpD* expression showed a 1.94-fold and 2-fold increase at 10 and 20 min, while *comEC* expression demonstrated a more modest elevation of 1.02-fold and 1.47-fold at 10 and 20 min, respectively. The expression trends for *dprA*, *cbpD*, and *comEC*, while not reaching statistical significance, suggest a temporal pattern of upregulation in response to CSP1 stimulation. Collectively, these results indicate that after the initiation of the competent development by CSP1 exposure, the expression of late *com* genes peaked at 20 min immediately after the peaked expression of the early *com* genes at 10 min (Figure 3B).

In order to compare to the competence development *in vitro* versus *in vivo* during pneumonic sepsis, re-analysis of some of our published RNA-Seq data (Oh et al., 2024) was necessary in order to provide a comprehensive comparison. In the RNA-Seq analysis, the expression profile of most of the late *com* genes is similar to that of the early *com* genes, with expression beginning to increase at 12-hpi and peaking at 24-hpi (Figure 3A). Notably, *cbpD* exhibited a distinct pattern, showing a significant peak at 12-hpi rather than at 24-hpi. This early expression of *cbpD in vivo* is consistent with its *in vitro* pattern, where expression was observed to initiate at 10 min post-CSP1 induction. These findings suggest that *cbpD* may be activated at an earlier time point compared to other late *com* genes, potentially indicating a unique role or regulation in the competence development process.

Next, we validated the reproducibility of *in vivo* expression of the late *com* genes, *cibA*, *dprA*, *ssbB*, *cbpD*, *comEC*, and *comGC*, using qPCR. The results confirmed the RNA-Seq findings that these late *com* genes exhibited upregulation from 12-hpi and peaked at 24-hpi, with the exception of *cbpD* (Figure 3A). Specifically, the expression of *dprA*, *ssbB*, and *comGC* showed a statistically significant increase in expression at 24-hpi when compared to baseline at 0-hpi (Figure 3A). Although *cibA*, *cbpD*, and *comEC* also displayed elevated expression at 12 or 24-hpi, the differences did not reach statistical significance when compared to baseline, primarily due to variability among samples. Despite the lack of statistical significance, notable fold changes in gene expression were observed for *cibA*, *cbpD*, and *comEC* relative to baseline (0-hpi). The expression of *cibA* increased 1.50-fold at 12-hpi and 2.23-fold at 24-hpi. Similarly, *cbpD* showed modest elevations of 1.36-fold and 1.15-fold at 12 and 24-hpi, respectively. The *comEC* genes exhibited the most pronounced change, with a 1.34-fold increase at 12-hpi followed by a substantial 3.85-fold upregulation at 24-hpi. Overall, an average gene expression of the selective late *com* genes displayed their highest expression at 24-hpi, as shown in the solid black line in Figure 3C. This expression pattern is similar to the expression observed for the selective early *com* genes in Figure 2C, suggesting a coordinated regulation of both early and late competence genes during pneumonic sepsis infection.

### Comparative analysis of the pneumococcal delayed *com* gene expression *in vitro* versus during pneumonic sepsis

Next, we examined the expression of delayed *com* genes *clpL*, *dnaK*, *gntR*, *groL*, *hrcA*, and *htrA*. The expression of *hrcA* showed a significant increase between 20 and 30 min post-CSP1 treatment, while *htrA* exhibited significant expression at 20 min. The expression levels of *dnaK* peaked at 30 min post-CSP1 exposure but were not statistically different from the Time 0 min baseline control. The expression of levels of *clpL*, *groL*, and *gntR* did not exhibit any significant changes across all experimental time points (Figure 4A). A potential explanation for this phenomenon could be that the delayed *com* genes are involved in stress responses (Santoro et al., 2019). In the absence of specific stressful stimuli during early log phase growth *in vitro*, particularly in the nutrient-rich THY broth, the gene expression levels for these delayed *com* genes remained low. A summary of the *in vitro* expression profiles for delayed competence genes is presented in Figure 4B. In contrast to the distinct expression peaks observed for early genes (at 10 min) and late genes (at 20 min) post-CSP1 treatment, the delayed genes did not exhibit a similarly pronounced peak in expression (Figures 4B, 5A). Instead, most delayed genes demonstrated modest and gradual changes in expression levels throughout the *in vitro* competent state period (Figure 4B).

Interestingly, reanalysis of our RNA-Seq data revealed that contrary to the aforementioned *in vitro* findings where all six delayed *com* genes were modestly upregulated during the competent state, the expression of *clpL*, *dnaK*, *groL*, and *hrcA* was repressed below the 0-hpi baseline levels during acute pneumonia-derived sepsis (Figure 4A). The expression of *gntR* and *htrA* was modestly increased at the 12-hpi and returned to near baseline levels between the 24-hpi and >40-hpi. Collectively, the delayed *com* genes were mostly downregulated during the course of pneumonic sepsis infection when compared to the 0-hpi (Figure 4C, solid black line).

The validation of the RNA-Seq results using RNA from the repeated new experiment by qPCR largely corroborated the gene expression patterns observed, with a few exceptions. Some genes (*dnaK* at 12, 24 and 40-hpi; both *groL* and *clpL* at 12-hpi) exhibited outliers in their expression profiles, which resulted in non-statistically significant differences when compared to 0-hpi. Despite these outliers, the overall trends in gene expression were consistent and reinforcing the validity of our transcriptomic analysis. Moreover, the expression profile of *htrA*, as revealed by RNA-Seq and subsequently validated through qPCR, exhibited a modest increased at 12-hpi but returned to near baseline levels between 24-hpi and beyond 40-hpi. Furthermore, the RNA-Seq analysis revealed a modest but sustained increase in *gntR* expression throughout the infection period. However, subsequent qPCR validation showed *gntR* expression levels falling below baseline at both 24 and 40-hpi. Interestingly, *gntR* expression pattern of slight increased at early time point during infection followed by decreased expression at later stages resembles the *gntR* expression profile observed *in vitro*. However, despite these observed trends, the changes in *gntR* expression did not reach statistical significance when compared to baseline level (Figure 4A).

### qPCR analysis validates the RNA-Seq transcriptomic profiles of competence genes with high fidelity

Collectively, our *in vitro* qPCR results recapitulated previously published microarray expression profiles of *com* genes (Peterson et al., 2004), where the selective early *com* genes displayed their highest expression at 10 min, followed by selective late *com* genes at 20 min, and the delayed *com* genes *hrcA* and *htrA* peaked between 20–30 min. A summarized representation of the *in vitro* study is shown in Figure 5A. Also, despite some discrepancies due to large error bars caused by outliers within some data sets, collectively, both *in vivo* RNA-Seq and qPCR results revealed that the expression of both early and late *com* genes was initiated at around 12-hpi and reached their peak expression at 24-hpi. In contrast, the expression of delayed genes was mostly suppressed throughout the acute pneumonia-derived sepsis (Figures 5B,C). A summarized representation of the *in vivo* study is shown in Figure 5D.

Importantly, our qPCR validation using RNA from our new pneumonic sepsis experiment aligns closely with the expression profiles as revealed by the RNA-Seq. A Pearson correlation coefficient of 0.7775 with a *p*-value of <0.0001 demonstrates a strong correlation between the RNA-Seq and qPCR expression data on the 18 *com* genes (Figure 5E). Each data point within the dataset represents the log_2_ fold change at either 12, 24, 40-hpi in relative to 0-hpi, resulting in a total of 54 values. To facilitate a clearer comparison, the expression of early, late, and delayed *com* genes were color-coded. As shown in Figure 5E, the majority of the early (red dots) and late (blue dots) *com* genes showed upregulation and localized to the first quadrant whereas the delayed *com* genes (green dots) are located in the third quadrant, indicating a down-regulation in expression. A detailed correlation graph with distinct gene symbols is plotted accordingly and can be found in Supplementary Figure S1. This alignment not only validates our transcriptomic findings but also reinforces the reliability of our gene expression analysis during pneumococcal infection.

## Discussion

The competent state in *S. pneumoniae* is a transient cellular phase that allows the uptake of environmental DNA for genetic recombination and fitness. This state involves the induction of three distinct phases of early, late, and delayed *com* genes (Lin et al., 2016; Johnston et al., 2014). In this study, we compared the expression profiles of six early, late, and delayed *com* genes during the *in vitro* CSP1-induced competent state versus the naturally developed competent state during pneumonic sepsis. Consistent with previous findings in the serotype 4 TIGR4 strain (Griffith, 1928), our *in vitro* qPCR-based studies in the serotype 2 strain D39 revealed that the expression of early *com* genes was induced the earliest at approximately 10 min post-CSP1 treatment. Following the activation of early *com* genes, the late *com* genes were subsequently expressed at approximately 20 min after induction with CSP1. The expression of the delayed genes was only modestly increased and peaked at 30 min, especially for *dnaK* and *hrcA*. During acute lung infection, the expression of both early and late *com* genes began before 12-hpi and reached their maximum at 24-hpi. However, the expression of early and late *com* genes was maintained until the mice became moribund and had to be euthanized. Importantly, the peaked expression of late competence genes, such as *cibA* and *cbpD*, which encode the allolytic autolysins responsible for pneumolysin release, was followed by the breach of the alveolar-capillary barrier and systemic invasion. A significant divergence in the gene expression profile between *in vitro* versus pneumonic sepsis was observed in the expression of delayed genes, which were downregulated throughout lung infection from Time 0-hpi to >40-hpi.

The delayed competence genes in *S. pneumoniae* encode proteins commonly associated with stress responses, including chaperones, proteases, and heat shock proteins (Rimini et al., 2000; Peterson et al., 2004; Dagkessamanskaia et al., 2004). Our *in vitro* competence induction studies revealed that the expression of delayed genes was modestly elevated, aligning with previous findings in *Streptococcus gordonii*, a streptococcus closely related taxonomically to *S. pneumoniae*. In *S. gordonii*, stress-associated genes were not upregulated within 40 min post-CSP1 treatment, and some delayed genes showed synchronized expression with late genes with a strong signal detected 15 min after CSP1 induction (Vickerman et al., 2007). This observation aligns with our findings on *hrcA* and *htrA* expression in *S. pneumoniae*, which peaked at 20 min post CSP1 induction. One intriguing aspect of our findings is the downregulation of the many delayed genes (*clpL*, *hrcA*, *dnaK*, *groL*) throughout the entire course of lung infection, which is different than the expression profiles of these genes during competence development *in vitro*, which were either constitutive or peaked at 20 to 30 min post-exposure to CSP1, and without significant downregulation thereafter (Figure 4). The expression levels of *gntR* and *htrA* in the infected lungs were not appreciably changed. The GntR-like transcriptional regulator (SPD_1524) has been shown to sense environmental and nutritional changes by regulating sugar transport and metabolism (Li et al., 2018) and glutamine/glutamate metabolism (Hendriksen et al., 2008), and is induced upon bacitracin and LL-37 exposures (Majchrzykiewicz et al., 2010). An increase in *gntR* expression at the initial stage could indeed be pivotal for virulence, particularly as bacteria metabolize sugar and amino acid such as glutamine and glutamate as crucial nutrients essential for their survival and pathogenicity (Paixão et al., 2015; Härtel et al., 2011). It is possible that by 12-hpi and beyond, D39 bacteria had adapted to the stressful lung environment to allow the expression of other genes important for pneumococcal pathogenesis. Lastly, the HtrA protease is a pneumococcal CSP1-degrading serine protease regulated by the CiaRH two-component regulatory system responsible for terminating the competent state (Attaiech et al., 2015; Cassone et al., 2012). The *htrA* gene was most highly expressed during the early stage of infection (12-hpi) followed by a gradual decline during the remaining course of pneumonic sepsis and was unable to shut down the competent state that seemed to be important for pneumococcal pathogenesis that lasted until the infected mice entered the moribund stage (Figure 1).

The observed patterns of competence gene expression *in vivo* likely reflect complex spatial and temporal dynamics within the bacterial population during infection. It is plausible that competence induction occurs in waves, propagating through different regions of the pneumococcal population asynchronously. This would result in a heterogeneous population where some subpopulations are undergoing competence while others are in the refractory period. The competence regulatory mechanisms elucidated through *in vitro* studies are likely still operational in the *in vivo* setting. However, the complex environment encountered during infection may lead to variations in competence induction and shut-off that are not captured by population-level transcriptomics.

Another point that required further clarification is that in both *in vitro* and *in vivo* studies, *S. pneumoniae* was cultured in THY broth. However, the preparation of inoculum for mouse infections was subjected to an additional step involving blood agar plating. This modification was implemented due to the tendency of *S. pneumoniae* to form long chains, potentially leading to an underestimation of the actual number of bacterial inoculum. To mitigate this inaccuracy, we incorporated a blood agar plating step in our inoculum preparation, as described in Lin et al. (2020) which resulted in formation of mostly diplococci typically seen in clinical settings. The pneumococcus cells were subjected to a 3 rounds of rigorous washing process using sterile saline solution. This procedure effectively removed any residual media components, and the pneumococcus cells were subsequently collected for infection. This approach allows for more accurate quantification and standardization of bacterial cells across experiments, and enhances the reproducibility and reliability of our *in vivo* infection model. Therefore, it is unlikely that differences in the nutritional aspects of THY versus blood agar caused the differences in competence development between *in vivo* and *in vitro* conditions.

Competence development in *S. pneumoniae* is a complex process highly regulated by multiple factors, including specific host tissue micro-environmental conditions, nutrient availability, and accumulation of CSP. Through normal daily breathing, we inhale millions of particles and microbes. Most of these harmful substances and microbes are trapped by the mucous layer coating upper airways and cleared by mucociliary transport. Microbes that successfully reach the alveolar spaces are deposited on the pulmonary surfactant layer, which initiates complex strategies to counter each other. Similarly, during the initial stages of lung infection, pneumococcus senses changes in host body temperature, pH, ionic strength, and viscosity within the surfactant layer, forcing it to adapt to the host environment to establish infection. In addition, pneumococcal cells would need to overcome various first-line host innate immune defences in the alveolar lining layer such as antimicrobial peptides (MacNair et al., 2024), surfactant proteins and phospholipids (Kuang et al., 2020; Kuang et al., 2011; Tan et al., 2014; Awasthi, 2010; Tan et al., 2015), cell wall-degrading lysozyme (Davis et al., 2008), lactoferrin (Bitsaktsis et al., 2012), secretory phospholipase A2 (Movert et al., 2013), secretory leukocyte protease inhibitor (Abe et al., 1997), as well as phagocytic alveolar macrophages and neutrophils (Koppe et al., 2012) and the complement system (Syed et al., 2020). Pneumococci likely need to overcome these first lines of host defences before establishing successful infection and competence development. Our recent RNA-Seq analysis revealed that when compared against Time 0-hpi (baseline), 20, 42, and 24% of the top 50 most upregulated pneumococcal genes in infected mouse lungs at 12-hpi, 24-hpi and >40-hpi were *com* genes (Oh et al., 2024), indicating crucial roles played by the competence regulon in initial adaptation to the lung environment (0–12-hpi), followed by lung infection and breaching of the air-blood barrier (12–24-hpi), and maintenance of the septicemic stage (24–40-hpi) (Oh et al., 2024). Nevertheless, early competence genes were the first to be expressed when the pneumococcus cells entered a competent state. As a key regulators of competence, the early genes *comCDE* play indispensable roles in regulating competence development (Martin et al., 2013), consequently, these genes exhibit drastically higher expression levels *in vitro*. Our study confirms that these genes exhibit significantly elevated expression levels 10 min after CSP1 treatment *in vitro* (Figure 2A). Specifically, *comC* shows peak expression at 10 min with a 9.06-fold increase, while *comD* and *comE* display even more dramatic upregulation with 122-fold and 38.8-fold increases, respectively. These results underscore the rapid and robust transcriptional response of the ComCDE system to CSP1 induction. Unexpectedly, the expression for *comD* and *comE* were barely changed at 12-hpi but upregulated at 24-hpi by 7.5-fold and continuously maintained much above the baseline level even at the moribund state, as reflected by persistent activation of the competent state based on our RNA-Seq data.

All the *in vitro* experiments in this study were performed in THY broth using the *S. pneumoniae* D39 strain. D39 does not enter the competent state naturally in THY broth without the provision of CSP1. Our lab has previously confirmed this using the reporter strain D39-*ssbB*-*luc* and tested its bioluminescence activity, finding that it failed to enter the competent state naturally in the THY medium. After the addition of CSP1, we were able to monitor bioluminescence activity 30 min (indicating expression of *ssbB* late gene) after the provision of CSP1 (Lin et al., 2020). During acute pneumonia infection in mice, the competent state developed naturally without the exogenous provision CSP1. However, the conditions that are required for the competence development is unknown. During the lung infection, pneumococci compromise the alveolar-capillary barrier, allowing pneumococcal cells to enter the systemic circulation. This breach not only facilitates the spread of infection but also creates conditions favourable for competence development. The lung environment typically low in certain nutrients, especially the divalent cations. Specifically, the damaged alveolar-capillary barrier allows the influx of various substances, including divalent cations such as calcium, magnesium, and manganese, which are usually scarce in lung tissue. Pneumococcal cells can then uptake these nutrients, which have been shown to be important for the induction of competence (Oh et al., 2024). This nutrient availability likely contributes to the natural induction of competence *in vivo*, even in the absence of exogenous CSP1. As we have reported previously, the synthetic C + Y medium consists various divalent metal ions which support spontaneous induction of competence in *S. pneumoniae* CP1250 strains at competence permissive pH (Oh et al., 2024).

In addition, our previous research has also demonstrated that the exogenous provision of CSP1 has limited effect on the development of natural competence during pneumonia-derived sepsis. Surprisingly, exogenously provided CSP1 failed to modulate the onset kinetics of competence development *in vivo*. Interestingly, our previous findings revealed that competent D39 bacteria primarily spread the competence signal through direct cell-to-cell contact. This mechanism diverges from the classical quorum-sensing model, which relies heavily on the accumulation of extracellular CSP (Lin et al., 2020).

RNA-Seq is an extremely sensitive technique widely used to provide a comprehensive transcriptional profile, capable of detecting even low-abundance transcripts. Our previous RNA-Seq analysis revealed a significant upregulation of competence genes in *S. pneumoniae* as early as 12-hpi during pneumonic sepsis in mice. This finding suggests that the pneumococcus competence regulon plays a crucial role in pneumococci adaptation to the lung microenvironment during infection. However, when we monitored the progression of infection using the IVIS system, we did not detect a competent state until approximately 24-hpi (e.g., compare Figure 1A vs. Figure 1C). Interestingly, both early and late competence genes reached their peak expression at 24-hpi, marking the peak competent state where the late genes tend to express in relatively rapid succession following the expression of early genes. The expression of the *ssbB* gene (luciferase signals) was detectable only at 24-hpi, perhaps reaching an expression threshold where could be detected by the less sensitive IVIS-based live imaging. Interestingly, the IVIS data shows the strongest *ssbB* bioluminescence signal at 40-hpi, which shows a discrepancy compared to the peaked *ssbB* expression at 24-hpi by RNA-Seq. The stronger IVIS signal observed at 40-hpi can be attributed to a cascade of events within the host. As the alveolar-capillary barrier was breached, bacteria gained access to the nutrient-rich bloodstream, allowing them to spread systemically rather than remain confined to the lungs. This widespread dissemination throughout the host body resulted in a more intense, systemic bioluminescence signal output at 40-hpi. Furthermore, the nutrient-rich blood created conditions more favorable for pneumococcal competence development, leading to an increase in competence signal. Another plausible explanation is that, the IVIS imaging and RNA-Seq employ different technologies to assess gene expression, which can lead to apparent discrepancies in results. IVIS detects bioluminescence signals from reporter proteins expressed by *S. pneumoniae*, while RNA-Seq directly quantifies mRNA levels. This fundamental difference can result in temporal variations in peak expression detection. The observed delayed peak in the IVIS signal at 40-hpi likely reflects post-transcriptional regulation of SsbB. Even after mRNA levels have decreased, the reporter protein can continue to accumulate, resulting in a sustained or increased bioluminescence signal. Therefore, mRNA transcriptome levels do not always directly correlate with protein levels. Also, the half-life mRNA molecules is generally much shorter than proteins. This discrepancy is further supported by findings from Cheng et al. (2016) who reported that while mRNA concentrations declined, protein concentrations continued to increase.

It is worth noting that, the initial competence induction in the context of pneumococcal cells relies more on the adaptation to the lung environment rather than the number of pneumococcal cells present (Lin et al., 2020; Weiser et al., 2018). Lin et al. (2020) demonstrated that regardless of whether mice were inoculated with 5 × 10^7^, 5 × 10^6^ or 1 × 10^6^ CFU, competence induction was consistently detected at approximately 24-hpi in all cases. This observation suggests that the ability of bacteria to sense and respond to the host environment, rather than their initial numbers, is the key trigger for competence induction. The consistency in timing across different inoculum sizes underscores the importance of bacterial adaptation to the host milieu in regulating this crucial physiological process. In addition, the authors also revealed that the lung bacterial burden had minimal impact on competence induction kinetics. The authors further explored this by infecting mice with 5 × 10^7^ CFU of D39-*ssbB*-*luc* and performed the bacterial burden at 0, 12, 24, and >40-hpi. They found no significant difference in pneumococcal lung burden at 12 and 24-hpi compared to 0-hpi, with a significant increase to >10^8^ CFU only after 40-hpi. Notably, initial competence induction at approximately 24-hpi appeared to depend more on adaptation to the lung environment rather than bacterial cell numbers (Lin et al., 2020).

Our previous study demonstrated a high degree of correlation between RNA-Seq and qPCR validation results for 10 representative competence-specific genes, using the same RNA samples. This analysis yielded a Pearson correlation coefficient *r* of 0.8525 (*R*^2^ = 0.7267) (Oh et al., 2024). In the current study, we expanded our analysis to 18 pneumococcal genes to further assess the reproducibility of the RNA-Seq data. Importantly, we utilized RNA extracted from a new independently performed pneumonic sepsis experiment. This expanded validation yielded a Pearson correlation coefficient *r* of 0.7775, further confirming the reliability of our RNA-Seq results. Minhas et al. (2020) employed the same approach to validate the reproducibility of their dual RNA-Seq data for both murine and pneumococcal genes. They used RNA isolated from two sources, the same isolated RNA for dual RNA-Seq and RNA from a repeated experiment. When using RNA from the same samples for dual RNA-Seq, they obtained a Pearson *r* of 0.8565 (*R*^2^ = 0.7338), while using isolated RNA from a repeated experiment resulted in Pearson *r* of 0.8751 (*R*^2^ = 0.7658) (Minhas et al., 2020). Overall, these results highlight the robustness and reproducibility of RNA-Seq data across different experimental conditions and biological systems. The high correlation between RNA-Seq and qPCR validation results, as well as the strong reproducibility demonstrated in both our studies and in Minhas et al. (2020) suggest that RNA-Seq provides reliable and consistent gene expression data. Importantly, this high level of reproducibility indicates that repeating RNA-Seq experiments may not be necessary in many cases, which is particularly advantageous given the considerable cost associated with RNA-Seq. This finding not only validates the reliability of RNA-Seq as a tool for gene expression analysis but also highlights its cost-effectiveness in experimental design.

In summary, a more comprehensive comparison of the similarities and differences of the gene expression patterns during *in vitro* and *in vivo* competence development may reveal new aspects of uncharacterized pneumococcal competence regulation. These new insights could contribute to the development of innovative strategies for controlling the burden of pneumococcal diseases by inhibiting the spread of virulence and antibiotic resistance genes mediated by the competence regulon.

## Data Availability

The datasets presented in this study can be found in online repositories. The names of the repository/repositories and accession number(s) can be found in the article/Supplementary material.

## References

[ref1] Abe T. Tominaga Y. Kikuchi T. Watanabe A. Satoh K. Watanabe Y.. (1997). Bacterial pneumonia causes augmented expression of the secretory leukoprotease inhibitor gene in the murine lung. Am. J. Respir. Crit. Care Med. 156, 1235–1240. doi: 10.1164/ajrccm.156.4.9701075, PMID: 9351627

[ref2] Alhamdi Y. Neill D. R. Abrams S. T. Malak H. A. Yahya R. Barrett-Jolley R.. (2015). Circulating pneumolysin is a potent inducer of cardiac injury during pneumococcal infection. PLoS Pathog. 11:e1004836. doi: 10.1371/journal.ppat.1004836, PMID: 25973949 PMC4431880

[ref3] Attaiech L. Minnen A. Kjos M. Gruber S. Veening J. W. (2015). The ParB-parS chromosome segregation system modulates competence development in *Streptococcus pneumoniae*. mBio 6:e00662. doi: 10.1128/mBio.00662-15, PMID: 26126852 PMC4488948

[ref4] Attaiech L. Olivier A. Mortier-Barrière I. Soulet A. L. Granadel C. Martin B.. (2011). Role of the single-stranded DNA-binding protein SsbB in pneumococcal transformation: maintenance of a reservoir for genetic plasticity. PLoS Genet. 7:e1002156. doi: 10.1371/journal.pgen.1002156, PMID: 21738490 PMC3128108

[ref5] Awasthi S. (2010). Surfactant protein (SP)-A and SP-D as antimicrobial and immunotherapeutic agents. Recent Pat. Antiinfect. Drug Discov. 5, 115–123. doi: 10.2174/157489110791233559, PMID: 20230362

[ref6] Bergé M. Mercy C. Mortier-Barrière I. VanNieuwenhze M. Brun Y. Grangeasse C.. (2017). A programmed cell division delay preserves genome integrity during natural genetic transformation in *Streptococcus pneumoniae*. Nat. Commun. 8:1621. doi: 10.1038/s41467-017-01716-9, PMID: 29158515 PMC5696345

[ref7] Bitsaktsis C. Iglesias B. V. Li Y. Colino J. Snapper C. M. Hollingshead S. K.. (2012). Mucosal immunization with an unadjuvanted vaccine that targets *Streptococcus pneumoniae* PspA to human Fcγ receptor type I protects against pneumococcal infection through complement- and lactoferrin-mediated bactericidal activity. Infect. Immun. 80, 1166–1180. doi: 10.1128/IAI.05511-11, PMID: 22158740 PMC3294663

[ref8] Brown A. O. Mann B. Gao G. Hankins J. S. Humann J. Giardina J.. (2014). *Streptococcus pneumoniae* translocates into the myocardium and forms unique microlesions that disrupt cardiac function. PLoS Pathog. 10:e1004383. doi: 10.1371/journal.ppat.1004383, PMID: 25232870 PMC4169480

[ref9] Cassone M. Gagne A. L. Spruce L. A. Seeholzer S. H. Sebert M. E. (2012). The HtrA protease from *Streptococcus pneumoniae* digests both denatured proteins and the competence-stimulating peptide. J. Biol. Chem. 287, 38449–38459. doi: 10.1074/jbc.M112.391482, PMID: 23012372 PMC3493890

[ref10] Cheng Z. Teo G. Krueger S. Rock T. M. Koh H. W. L. Choi H.. (2016). Differential dynamics of the mammalian mRNA and protein expression response to misfolding stress. Mol. Syst. Biol. 12:855. doi: 10.15252/msb.20156423, PMID: 26792871 PMC4731011

[ref11] Claverys J. P. Martin B. Polard P. (2009). The genetic transformation machinery: composition, localization, and mechanism. FEMS Microbiol. Rev. 33, 643–656. doi: 10.1111/j.1574-6976.2009.00164.x, PMID: 19228200

[ref12] Dagkessamanskaia A. Moscoso M. Hénard V. Guiral S. Overweg K. Reuter M.. (2004). Interconnection of competence, stress and CiaR regulons in *Streptococcus pneumoniae*: competence triggers stationary phase autolysis of ciaR mutant cells. Mol. Microbiol. 51, 1071–1086. doi: 10.1111/j.1365-2958.2003.03892.x, PMID: 14763981

[ref13] Davis K. M. Akinbi H. T. Standish A. J. Weiser J. N. (2008). Resistance to mucosal lysozyme compensates for the fitness deficit of peptidoglycan modifications by *Streptococcus pneumoniae*. PLoS Pathog. 4:e1000241. doi: 10.1371/journal.ppat.1000241, PMID: 19079576 PMC2587705

[ref14] Eldholm V. Johnsborg O. Straume D. Ohnstad H. S. Berg K. H. Hermoso J. A.. (2010). Pneumococcal CbpD is a murein hydrolase that requires a dual cell envelope binding specificity to kill target cells during fratricide. Mol. Microbiol. 76, 905–917. doi: 10.1111/j.1365-2958.2010.07143.x, PMID: 20384696

[ref15] Feikin D. R. Kagucia E. W. Loo J. D. Link-Gelles R. Puhan M. A. Cherian T.. (2013). Serotype-specific changes in invasive pneumococcal disease after pneumococcal conjugate vaccine introduction: a pooled analysis of multiple surveillance sites. PLoS Med. 10:e1001517. doi: 10.1371/journal.pmed.1001517, PMID: 24086113 PMC3782411

[ref16] Feldman C. Anderson R. (2014). Review: current and new generation pneumococcal vaccines. J. Infect. 69, 309–325. doi: 10.1016/j.jinf.2014.06.006, PMID: 24968238

[ref17] Feldman C. Anderson R. (2016). The role of *Streptococcus pneumoniae* in community-acquired pneumonia. Semin. Respir. Crit. Care Med. 37, 806–818. doi: 10.1055/s-0036-1592074, PMID: 27960205

[ref18] Gadsby N. J. Musher D. M. (2022). The microbial etiology of community-acquired pneumonia in adults: from classical bacteriology to host transcriptional signatures. Clin. Microbiol. Rev. 35:e0001522. doi: 10.1128/cmr.00015-22, PMID: 36165783 PMC9769922

[ref19] Gagne A. L. Stevens K. E. Cassone M. Pujari A. Abiola O. E. Chang D. J.. (2013). Competence in *Streptococcus pneumoniae* is a response to an increasing mutational burden. PLoS One 8:e72613. doi: 10.1371/journal.pone.0072613, PMID: 23967325 PMC3742669

[ref20] Geno K. A. Gilbert G. L. Song J. Y. Skovsted I. C. Klugman K. P. Jones C.. (2015). Pneumococcal capsules and their types: past, present, and future. Clin. Microbiol. Rev. 28, 871–899. doi: 10.1128/CMR.00024-15, PMID: 26085553 PMC4475641

[ref21] Griffith F. (1928). The significance of pneumococcal types. J. Hyg. 27, 113–159. doi: 10.1017/s0022172400031879, PMID: 20474956 PMC2167760

[ref22] Guiral S. Henard V. Granadel C. Martin B. Claverys J. P. (2006). Inhibition of competence development in *Streptococcus pneumoniae* by increased basal-level expression of the ComDE two-component regulatory system. Microbiology 152, 323–331. doi: 10.1099/mic.0.28425-0, PMID: 16436420

[ref23] Härtel T. Klein M. Koedel U. Rohde M. Petruschka L. Hammerschmidt S. (2011). Impact of glutamine transporters on pneumococcal fitness under infection-related conditions. Infect. Immun. 79, 44–58. doi: 10.1128/IAI.00855-10, PMID: 21078855 PMC3019899

[ref24] Havarstein L. S. Coomaraswamy G. Morrison D. A. (1995). An unmodified heptadecapeptide pheromone induces competence for genetic transformation in *Streptococcus pneumoniae*. Proc. Natl. Acad. Sci. U.S.A. 92, 11140–11144. doi: 10.1073/pnas.92.24.11140, PMID: 7479953 PMC40587

[ref25] Håvarstein L. S. Gaustad P. Nes I. F. Morrison D. A. (1996). Identification of the streptococcal competence-pheromone receptor. Mol. Microbiol. 21, 863–869. doi: 10.1046/j.1365-2958.1996.521416.x, PMID: 8878047

[ref26] Havarstein L. S. Martin B. Johnsborg O. Granadel C. Claverys J. P. (2006). New insights into the pneumococcal fratricide: relationship to clumping and identification of a novel immunity factor. Mol. Microbiol. 59, 1297–1037. doi: 10.1111/j.1365-2958.2005.05021.x, PMID: 16430701

[ref27] Hendriksen W. T. Kloosterman T. G. Bootsma H. J. Estevão S. de Groot R. Kuipers O. P.. (2008). Site-specific contributions of glutamine-dependent regulator GlnR and GlnR-regulated genes to virulence of *Streptococcus pneumoniae*. Infect. Immun. 76, 1230–1238. doi: 10.1128/IAI.01004-07, PMID: 18174343 PMC2258823

[ref28] Jaiswal N. Singh M. Das R. R. Jindal I. Agarwal A. Thumburu K. K.. (2014). Distribution of serotypes, vaccine coverage, and antimicrobial susceptibility pattern of *Streptococcus pneumoniae* in children living in SAARC countries: a systematic review. PLoS One 9:e108617. doi: 10.1371/journal.pone.0108617, PMID: 25268974 PMC4182530

[ref29] Johnsborg O. Havarstein L. S. (2009). Regulation of natural genetic transformation and acquisition of transforming DNA in *Streptococcus pneumoniae*. FEMS Microbiol. Rev. 33, 627–642. doi: 10.1111/j.1574-6976.2009.00167.x, PMID: 19396959

[ref30] Johnson C. N. Wilde S. Tuomanen E. Rosch J. W. (2024). Convergent impact of vaccination and antibiotic pressures on pneumococcal populations. Cell Chem. Biol. 31, 195–206. doi: 10.1016/j.chembiol.2023.11.003, PMID: 38052216 PMC10938186

[ref31] Johnston C. Martin B. Fichant G. Polard P. Claverys J. P. (2014). Bacterial transformation: distribution, shared mechanisms and divergent control. Nat. Rev. Microbiol. 12, 181–196. doi: 10.1038/nrmicro3199, PMID: 24509783

[ref32] Kjos M. Miller E. Slager J. Lake F. B. Gericke O. Roberts I. S.. (2016). Expression of *Streptococcus pneumoniae* bacteriocins is induced by antibiotics via regulatory interplay with the competence system. PLoS Pathog. 12:e1005422. doi: 10.1371/journal.ppat.1005422, PMID: 26840404 PMC4739728

[ref33] Koppe U. Suttorp N. Opitz B. (2012). Recognition of *Streptococcus pneumoniae* by the innate immune system. Cell. Microbiol. 14, 460–466. doi: 10.1111/j.1462-5822.2011.01746.x, PMID: 22212419

[ref34] Kuang Z. Bennett R. C. Lin J. Hao Y. Zhu L. Akinbi H. T.. (2020). Surfactant phospholipids act as molecular switches for premature induction of quorum sensing-dependent virulence in *Pseudomonas aeruginosa*. Virulence 11, 1090–1107. doi: 10.1080/21505594.2020.1809327, PMID: 32842850 PMC7549932

[ref35] Kuang Z. Hao Y. Walling B. E. Jeffries J. L. Ohman D. E. Lau G. W. (2011). *Pseudomonas aeruginosa* elastase provides an escape from phagocytosis by degrading the pulmonary surfactant protein-A. PLoS One 6:e27091. doi: 10.1371/journal.pone.0027091, PMID: 22069491 PMC3206073

[ref36] Lau G. W. Haataja S. Lonetto M. Kensit S. E. Marra A. Bryant A. P.. (2001). A functional genomic analysis of type 3 *Streptococcus pneumoniae* virulence. Mol. Microbiol. 40, 555–571. doi: 10.1046/j.1365-2958.2001.02335.x 11359563

[ref37] Laurenceau R. Krasteva P. V. Diallo A. Ouarti S. Duchateau M. Malosse C.. (2015). Conserved *Streptococcus pneumoniae* spirosomes suggest a single type of transformation pilus in competence. PLoS Pathog. 11:e1004835. doi: 10.1371/journal.ppat.1004835, PMID: 25876066 PMC4398557

[ref38] Lee M. S. Morrison D. A. (1999). Identification of a new regulator in *Streptococcus pneumoniae* linking quorum sensing to competence for genetic transformation. J. Bacteriol. 181, 5004–5016. doi: 10.1128/jb.181.16.5004-5016.1999, PMID: 10438773 PMC93990

[ref39] Li L. Ma J. Yu Z. Li M. Zhang W. Sun H. (2023). Epidemiological characteristics and antibiotic resistance mechanisms of *Streptococcus pneumoniae*: an updated review. Microbiol. Res. 266:127221. doi: 10.1016/j.micres.2022.127221, PMID: 36244081

[ref40] Li Z. Xiang Z. Zeng J. Li Y. Li J. (2018). A GntR family transcription factor in *Streptococcus mutans* regulates biofilm formation and expression of multiple sugar transporter genes. Front. Microbiol. 9:3224. doi: 10.3389/fmicb.2018.03224, PMID: 30692967 PMC6340165

[ref41] Lin J. Lau G. W. (2019). DprA-dependent exit from the competent state regulates multifaceted *Streptococcus pneumoniae* virulence. Infect. Immun. 87:e00349. doi: 10.1128/IAI.00349-19, PMID: 31451619 PMC6803342

[ref42] Lin J. Park P. Li H. Oh M. Dobrucki I. Dobrucki W.. (2020). *Streptococcus pneumoniae* elaborates persistent and prolonged competent state during pneumonia-derived sepsis. Infect. Immun. 88:e00919. doi: 10.1128/iai.00919-19, PMID: 31988172 PMC7093142

[ref43] Lin J. Zhu L. Lau G. W. (2016). Disentangling competence for genetic transformation and virulence in *Streptococcus pneumoniae*. Curr. Genet. 62, 97–103. doi: 10.1007/s00294-015-0520-z, PMID: 26403231

[ref44] Luo P. Li H. Morrison D. A. (2003). ComX is a unique link between multiple quorum sensing outputs and competence in *Streptococcus pneumoniae*. Mol. Microbiol. 50, 623–633. doi: 10.1046/j.1365-2958.2003.03714.x, PMID: 14617184

[ref45] Luo P. Morrison D. A. (2003). Transient association of an alternative sigma factor, ComX, with RNA polymerase during the period of competence for genetic transformation in *Streptococcus pneumoniae*. J. Bacteriol. 185, 349–358. doi: 10.1128/jb.185.1.349-358.2003, PMID: 12486073 PMC141820

[ref46] MacNair C. R. Rutherford S. T. Tan M. W. (2024). Alternative therapeutic strategies to treat antibiotic-resistant pathogens. Nat. Rev. Microbiol. 22, 262–275. doi: 10.1038/s41579-023-00993-0, PMID: 38082064

[ref47] Majchrzykiewicz J. A. Kuipers O. P. Bijlsma J. J. (2010). Generic and specific adaptive responses of *Streptococcus pneumoniae* to challenge with three distinct antimicrobial peptides, bacitracin, LL-37, and nisin. Antimicrob. Agents Chemother. 54, 440–451. doi: 10.1128/AAC.00769-09, PMID: 19917758 PMC2798553

[ref48] Manna S. Spry L. Wee-Hee A. Ortika B. D. Boelsen L. K. Batinovic S.. (2022). Variants of *Streptococcus pneumoniae* serotype 14 from Papua New Guinea with the potential to be mistyped and escape vaccine-induced protection. Microbiol. Spectr. 10:e0152422. doi: 10.1128/spectrum.01524-22, PMID: 35862970 PMC9431120

[ref49] Manna S. Waring A. Papanicolaou A. Hall N. E. Bozinovski S. Dunne E. M.. (2018). The transcriptomic response of *Streptococcus pneumoniae* following exposure to cigarette smoke extract. Sci. Rep. 8:15716. doi: 10.1038/s41598-018-34103-5, PMID: 30356075 PMC6200755

[ref50] Martin B. Soulet A. L. Mirouze N. Prudhomme M. Mortier-Barrière I. Granadel C.. (2013). ComE/ComE~P interplay dictates activation or extinction status of pneumococcal X-state (competence). Mol. Microbiol. 87, 394–411. doi: 10.1111/mmi.12104, PMID: 23216914

[ref51] Mehr S. Wood N. (2012). *Streptococcus pneumoniae*—a review of carriage, infection, serotype replacement and vaccination. Paediatr. Respir. Rev. 13, 258–264. doi: 10.1016/j.prrv.2011.12.001, PMID: 23069126

[ref52] Minhas V. Aprianto R. McAllister L. J. Wang H. David S. C. McLean K. T.. (2020). *In vivo* dual RNA-seq reveals that neutrophil recruitment underlies differential tissue tropism of *Streptococcus pneumoniae*. Commun. Biol. 3:293. doi: 10.1038/s42003-020-1018-x, PMID: 32504007 PMC7275033

[ref53] Mirouze N. Bergé M. A. Soulet A. L. Mortier-Barrière I. Quentin Y. Fichant G.. (2013). Direct involvement of DprA, the transformation-dedicated RecA loader, in the shut-off of pneumococcal competence. Proc. Natl. Acad. Sci. U.S.A. 110, E1035–E1044. doi: 10.1073/pnas.1219868110, PMID: 23440217 PMC3600483

[ref54] Mortier-Barrière I. Velten M. Dupaigne P. Mirouze N. Piétrement O. McGovern S.. (2007). A key presynaptic role in transformation for a widespread bacterial protein: DprA conveys incoming ssDNA to RecA. Cell 130, 824–836. doi: 10.1016/j.cell.2007.07.038, PMID: 17803906

[ref55] Movert E. Wu Y. Lambeau G. Kahn F. Touqui L. Areschoug T. (2013). Secreted group IIA phospholipase A2 protects humans against the group B streptococcus: experimental and clinical evidence. J. Infect. Dis. 208, 2025–2035. doi: 10.1093/infdis/jit359, PMID: 23901095

[ref56] Nikolaou E. Hubbard A. T. M. Botelho J. Marschall T. A. M. Ferreira D. M. Roberts A. P. (2020). Antibiotic resistance is associated with integrative and conjugative elements and Genomic Islands in naturally circulating *Streptococcus pneumoniae* isolates from adults in Liverpool, UK. Genes 11:625. doi: 10.3390/genes11060625, PMID: 32517221 PMC7348760

[ref57] Oh M. W. Lin J. Chong S. Y. Lew S. Q. Alam T. Lau G. W. (2024). Time-resolved RNA-seq analysis to unravel the *in vivo* competence induction by *Streptococcus pneumoniae* during pneumonia-derived sepsis. Microbiol. Spectr. 12:e03050. doi: 10.1128/spectrum.03050-23, PMID: 38305162 PMC10913500

[ref58] Olson G. Davis A. M. (2020). Diagnosis and treatment of adults with community-acquired pneumonia. JAMA 323, 885–886. doi: 10.1001/jama.2019.21118, PMID: 32027358

[ref59] Paixão L. Caldas J. Kloosterman T. Kuipers O. Vinga S. Neves A. (2015). Transcriptional and metabolic effects of glucose on *Streptococcus pneumoniae* sugar metabolism. Front. Microbiol. 6:1041. doi: 10.3389/fmicb.2015.01041, PMID: 26500614 PMC4595796

[ref60] Paton J. C. Trappetti C. (2019). *Streptococcus pneumoniae* capsular polysaccharide. Microbiol. Spectr. 7. doi: 10.1128/microbiolspec.GPP3-0019-2018, PMID: 30977464 PMC11590643

[ref61] Pestova E. Håvarstein L. Morrison D. (1996). Regulation of competence for genetic transformation in *Streptococcus pneumoniae* by an auto-induced peptide pheromone and a two-component regulatory system. Mol. Microbiol. 21, 853–862. doi: 10.1046/j.1365-2958.1996.501417.x, PMID: 8878046

[ref62] Peterson S. Cline R. T. Tettelin H. Sharov V. Morrison D. A. (2000). Gene expression analysis of the *Streptococcus pneumoniae* competence regulons by use of DNA microarrays. J. Bacteriol. 182, 6192–6202. doi: 10.1128/JB.182.21.6192-6202.2000, PMID: 11029442 PMC94756

[ref63] Peterson S. N. Sung C. K. Cline R. Desai B. V. Snesrud E. C. Luo P.. (2004). Identification of competence pheromone responsive genes in *Streptococcus pneumoniae* by use of DNA microarrays. Mol. Microbiol. 51, 1051–1070. doi: 10.1046/j.1365-2958.2003.03907.x, PMID: 14763980

[ref64] Pettigrew M. M. Marks L. R. Kong Y. Gent J. F. Roche-Hakansson H. Hakansson A. P. (2014). Dynamic changes in the *Streptococcus pneumoniae* transcriptome during transition from biofilm formation to invasive disease upon influenza a virus infection. Infect. Immun. 82, 4607–4619. doi: 10.1128/IAI.02225-14, PMID: 25135685 PMC4249342

[ref65] Piotrowski A. Luo P. Morrison D. A. (2009). Competence for genetic transformation in *Streptococcus pneumoniae*: termination of activity of the alternative sigma factor ComX is independent of proteolysis of ComX and ComW. J. Bacteriol. 191, 3359–3366. doi: 10.1128/JB.01750-08, PMID: 19286798 PMC2687157

[ref66] Price K. E. Camilli A. (2009). Pneumolysin localizes to the cell wall of *Streptococcus pneumoniae*. J. Bacteriol. 191, 2163–2168. doi: 10.1128/JB.01489-08, PMID: 19168620 PMC2655535

[ref67] Rimini R. Jansson B. Feger G. Roberts T. C. de Francesco M. Gozzi A.. (2000). Global analysis of transcription kinetics during competence development in *Streptococcus pneumoniae* using high density DNA arrays. Mol. Microbiol. 36, 1279–1292. doi: 10.1046/j.1365-2958.2000.01931.x, PMID: 10931279

[ref68] Salvadori G. Junges R. Morrison D. A. Petersen F. C. (2019). Competence in *Streptococcus pneumoniae* and close commensal relatives: mechanisms and implications. Front. Cell. Infect. Microbiol. 9:94. doi: 10.3389/fcimb.2019.00094, PMID: 31001492 PMC6456647

[ref69] Santoro F. Iannelli F. Pozzi G. (2019). Genomics and genetics of *Streptococcus pneumoniae*. Microbiol. Spectr. 7. doi: 10.1128/microbiolspec.GPP3-0025-2018, PMID: 31111814 PMC11315030

[ref70] Steinmoen H. Knutsen E. Havarstein L. S. (2002). Induction of natural competence in *Streptococcus pneumoniae* triggers lysis and DNA release from a subfraction of the cell population. Proc. Natl. Acad. Sci. U.S.A. 99, 7681–7686. doi: 10.1073/pnas.112464599, PMID: 12032343 PMC124321

[ref71] Stevens K. E. Chang D. Zwack E. E. Sebert M. E. (2011). Competence in *Streptococcus pneumoniae* is regulated by the rate of ribosomal decoding errors. mBio 2:e00071. doi: 10.1128/mBio.00071-11, PMID: 21933920 PMC3175624

[ref72] Syed S. Viazmina L. Mager R. Meri S. Haapasalo K. (2020). Streptococci and the complement system: interplay during infection, inflammation and autoimmunity. FEBS Lett. 594, 2570–2585. doi: 10.1002/1873-3468.13872, PMID: 32594520

[ref73] Tan R. M. Kuang Z. Hao Y. Lau G. W. (2014). Type IV pilus of *Pseudomonas aeruginosa* confers resistance to antimicrobial activities of the pulmonary surfactant protein-A. J. Innate Immun. 6, 227–239. doi: 10.1159/000354304, PMID: 24080545 PMC3944384

[ref74] Tan R. M. Kuang Z. Hao Y. Lee F. Lee T. Lee R. J.. (2015). Type IV pilus glycosylation mediates resistance of *Pseudomonas aeruginosa* to opsonic activities of the pulmonary surfactant protein a. Infect. Immun. 83, 1339–1346. doi: 10.1128/IAI.02874-14, PMID: 25605768 PMC4363409

[ref75] Throup J. P. Koretke K. K. Bryant A. P. Ingraham K. A. Chalker A. F. Ge Y.. (2000). A genomic analysis of two-component signal transduction in *Streptococcus pneumoniae*. Mol. Microbiol. 35, 566–576. doi: 10.1046/j.1365-2958.2000.01725.x, PMID: 10672179

[ref76] Vickerman M. M. Iobst S. Jesionowski A. M. Gill S. R. (2007). Genome-wide transcriptional changes in *Streptococcus gordonii* in response to competence signaling peptide. J. Bacteriol. 189, 7799–7807. doi: 10.1128/JB.01023-07, PMID: 17720781 PMC2168715

[ref77] Weiser J. N. Ferreira D. M. Paton J. C. (2018). *Streptococcus pneumoniae*: transmission, colonization and invasion. Nat. Rev. Microbiol. 16, 355–367. doi: 10.1038/s41579-018-0001-8, PMID: 29599457 PMC5949087

[ref78] Weyder M. Prudhomme M. Berge M. Polard P. Fichant G. (2018). Dynamic modeling of *Streptococcus pneumoniae* competence provides regulatory mechanistic insights into its tight temporal regulation. Front. Microbiol. 9:1637. doi: 10.3389/fmicb.2018.01637, PMID: 30087661 PMC6066662

[ref79] Zafar M. A. Hamaguchi S. Zangari T. Cammer M. Weiser J. N. (2017). Capsule type and amount affect shedding and transmission of *Streptococcus pneumoniae*. mBio 8:e00989. doi: 10.1128/mBio.00989-17, PMID: 28830943 PMC5565965

[ref80] Zafar M. A. Wang Y. Hamaguchi S. Weiser J. N. (2017). Host-to-host transmission of *Streptococcus pneumoniae* is driven by its inflammatory toxin, pneumolysin. Cell Host Microbe 21, 73–83. doi: 10.1016/j.chom.2016.12.005, PMID: 28081446 PMC5267320

[ref81] Zhu L. Lin J. Kuang Z. Vidal J. E. Lau G. W. (2015). Deletion analysis of *Streptococcus pneumoniae* late competence genes distinguishes virulence determinants that are dependent or independent of competence induction. Mol. Microbiol. 97, 151–165. doi: 10.1111/mmi.13016, PMID: 25846124 PMC4536566

