# Supplementary material for: Comparative analysis of the Streptococcus pneumoniae competence development in vitro versus in vivo during pneumonia-derived sepsis
Source: Front Microbiol. 2025 Jan 28;16:1540511. doi: 10.3389/fmicb.2025.1540511 (PMC11811101; doi:10.3389/fmicb.2025.1540511)
Supplement: Supplementary file 1 [file Data_Sheet_1.docx]

**Supplementary Materials for**

**Comparative analysis of the *Streptococcus pneumoniae* competence development *in vitro* versus *in vivo* during pneumonia-derived sepsis**

Sook Yin Chong, Shi Qian Lew, Tauqeer Alam, Christopher A. Gaulke, Gee W. Lau*

Department of Pathobiology, University of Illinois at Urbana-Champaign, Urbana, Illinois, 61802.

*To whom correspondence may be addressed. Gee W. Lau, [geelau@illinois.edu](mailto:geelau@illinois.edu); Address: 2001 South Lincoln Avenue, Urbana, Illinois, 61802.

**This PDF file includes:**

Table S1

Table S2

Figure S1

**Table S1.** Selective competence (*com*) responsive genes and their functions.

|  | **Locus Tag** | **Gene Name** | **Product** |
| --- | --- | --- | --- |
| **Early**  **Genes** | SPD_RS00235 | *comA* | Peptide cleavage/export ABC transporter ComA |
|  | SPD_RS00240 | *comB* | Competence pheromone export protein ComB |
|  | SPD_RS10930 | *comC* | Competence-stimulating peptide ComC |
|  | SPD_RS10925 | *comD* | Competence system sensor histidine kinase ComD |
|  | SPD_RS10920 | *comE* | Competence system response regulator ComE |
|  | SPD_RS02550 | *blpY* | Bacteriocin immunity protein BlpY |
| **Late**  **Genes** | SPD_RS00740 | *cibA* | Fratricide two-peptide bacteriocin subunit CibA |
|  | SPD_RS05990 | *dprA* | DNA-processing protein DprA |
|  | SPD_RS09110 | *ssbB* | Single-stranded DNA-binding protein SsbB |
|  | SPD_RS10730 | *cbpD* | Choline binding-anchored murein hydrolase CbpD |
|  | SPD_RS04540 | *comEC* | Late competence DNA transporter ComEC |
|  | SPD_RS09885 | *comGC* | Late competence pilus protein ComGC |
| **Delayed**  **Genes** | SPD_RS01685 | *clpL* | Putative ATP-dependent Clp proteinase ATP-binding subunit ClpL |
|  | SPD_RS02475 | *dnaK* | Molecular chaperone DnaK |
|  | SPD_RS08105 | *gntR* | GntR family transcriptional regulator |
|  | SPD_RS09100 | *groL* | Heat shock protein 60 family chaperone GroEL |
|  | SPD_RS02465 | *hrcA* | Heat-inducible transcriptional repressor HrcA |
|  | SPD_RS10945 | *htrA* | Serine protease, DegP/HtrA |

**Table S2**. List of primers used for qPCR.

|  | **Locus Tag** | **Gene Name** | **Forward Primer (5'-3')** | **Reverse Primer (5'-3')** |
| --- | --- | --- | --- | --- |
| **Early**  **Genes** | SPD_RS00235 | *comA* | CAGGTGGTCAACGTCAGAGA | CAGCAATAGTCAAGCGGTGA |
|  | SPD_RS00240 | *comB* | AATCGGCAACCTCATCAGTC | CCTCGCCCTGAGACTTGTAG |
|  | SPD_RS10930 | *comC* | CAACCTCATCTCCCCACCT | CAGTTTGTAGCTTTGAAGGAAAAAG |
|  | SPD_RS10925 | *comD* | CACCGTCACAACGAAAAAGA | TCGATGGATGAAACAGTATGAGA |
|  | SPD_RS10920 | *comE* | GAGTCGCAAACTCTGATCGAC | CAAGACAACGGGAAAAGTCC |
|  | SPD_RS02550 | *blpY* | CATTCGCGATTTGGCCTTGA | AACGGGAAGGTCGCTACAAT |
| **Late**  **Genes** | SPD_RS00740 | *cibA* | AGCTGCCAAACCAGAACCTA | AGATATTGATGGCGGTCTCG |
|  | SPD_RS05990 | *dprA* | GAACGCATCTTAGCCTCTGC | TCATTTTCCTGCCCGTAATC |
|  | SPD_RS09110 | *ssbB* | TACGCAATTCTCCATCAACG | AAAGACCAAAACGGTGAACG |
|  | SPD_RS10730 | *cbpD* | GTAGCCATCCACCGTCGTAT | CTGGTGAAATGCAGACAGGA |
|  | SPD_RS04540 | *comEC* | CTACTGGCTCAACATGGGGT | GACAAGACTCCTCCTGCTGT |
|  | SPD_RS09885 | *comGC* | GGCTTTCCACCACCTTAACA | ATCAGCGTGCTTTTCTTGCT |
| **Delayed Genes** | SPD_RS01685 | *clpL* | GTCGTAACCGTGCTGGTTTT | AGCAAGCTCCGTCTTACCAA |
|  | SPD_RS02475 | *dnaK* | AAACGCTGAAGCCGATAAGA | TTGCTTTCATGTCGTCCAAG |
|  | SPD_RS08105 | *gntR* | TACAGCAAGCGAACAACTGG | ATAGCCAAAATGGGTCATGG |
|  | SPD_RS09100 | *groL* | AAGATGCCCTCAACGCTACT | GAGCAATTTGACGAACGGGT |
|  | SPD_RS02465 | *hrcA* | GCTCATACTTCAAGCGGTCG | CACTACCGTACAGCCACTCA |
|  | SPD_RS10945 | *htrA* | GTTTCGCAATTCCTGCAAAT | CCGAACGAACAATTACACCA |
| **Reference Gene** | SPD_RS05765 | *gyrA* | CCATTTTGGATAGCGAGCAT | GGAAAAACGCATTGAGGGTA |


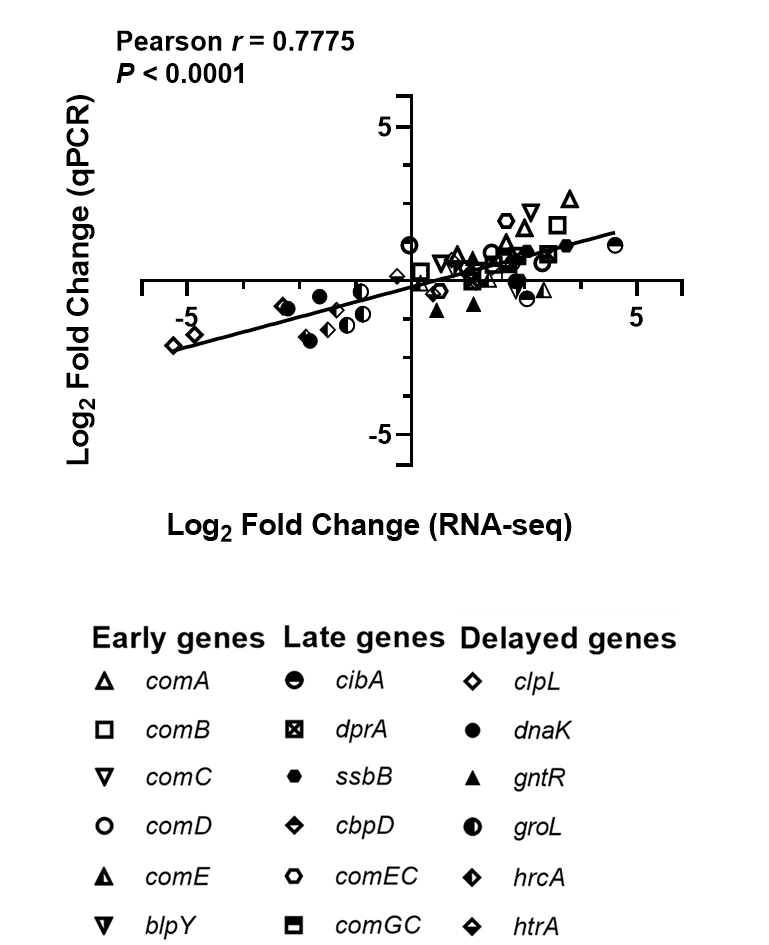


**S1**

**Figure S1**. Reproducibility validation of transcriptomic data with qPCR. Log_2_ fold changes in the expression of competence (*com*) genes from qPCR were plotted against RNA-Seq data. A total of 18 selective *com* genes were analyzed, with each symbol representing the log_2_ fold change at 12, 24, and > 40-hpi relative to 0-hpi, resulting in a total of 54 plotted values. A high degree of correlation was observed with Pearson *r* = 0.7775 (*p* < 0.0001). X-axis: Log_2_ Fold Change (RNA-Seq); Y-axis: Log_2_ Fold Change (qPCR).
